# β‐1,4‐Cellobiohydrolase is involved in full expression of *phcA*, contributing to the feedback loop in quorum sensing of *Ralstonia pseudosolanacearum* strain OE1‐1

**DOI:** 10.1111/mpp.13322

**Published:** 2023-03-13

**Authors:** Wakana Senuma, Masayuki Tsuzuki, Chika Takemura, Yuki Terazawa, Kanako Inoue, Akinori Kiba, Kouhei Ohnishi, Kenji Kai, Yasufumi Hikichi

**Affiliations:** ^1^ Faculty of Agriculture and Marine Science Kochi University Nankoku Japan; ^2^ Research Center for Ultra‐High Voltage Electron Microscopy Osaka University Ibaraki Japan; ^3^ Graduate School of Agriculture Osaka Metropolitan University Sakai Japan; ^4^ Present address: Central Research Institute, Ishihara Sangyo Ltd Kusatsu Japan; ^5^ Present address: Kochi Prefectural Agriculture Research Center Nankoku Japan

**Keywords:** *phcA*, quorum sensing, *Ralstonia pseudosolanacearum*, virulence, β‐1,4‐cellobiohydrolase

## Abstract

After infecting roots of tomato plants, the gram‐negative bacterium *Ralstonia pseudosolanacearum* strain OE1‐1 activates quorum sensing (QS) to induce production of plant cell wall‐degrading enzymes, such as β‐1,4‐endoglucanase (Egl) and β‐1,4‐cellobiohydrolase (CbhA), via the LysR family transcriptional regulator PhcA and then invades xylem vessels to exhibit virulence. The *phcA*‐deletion mutant (Δ*phcA*) exhibits neither the ability to infect xylem vessels nor virulence. Compared with strain OE1‐1, the *egl*‐deletion mutant (Δ*egl*) exhibits lower cellulose degradation activity, lower infectivity in xylem vessels, and reduced virulence. In this study, we analysed functions of CbhA other than cell wall degradation activity that are involved in the virulence of strain OE1‐1. The *cbhA*‐deletion mutant (Δ*cbhA*) lacked the ability to infect xylem vessels and displayed loss of virulence, similar to Δ*phcA*, but exhibited less reduced cellulose degradation activity compared with Δ*egl*. Transcriptome analysis revealed that the *phcA* expression levels in Δ*cbhA* were significantly lower than in OE1‐1, with significantly altered expression of more than 50% of PhcA‐regulated genes. Deletion of *cbhA* led to a significant change in QS‐dependent phenotypes, similar to the effects of *phcA* deletion. Complementation of Δ*cbhA* with native *cbhA* or transformation of this mutant with *phcA* controlled by a constitutive promoter recovered its QS‐dependent phenotypes. The expression level of *phcA* in Δ*cbhA*‐inoculated tomato plants was significantly lower than in strain OE1‐1‐inoculated plants. Our results collectively suggest that CbhA is involved in the full expression of *phcA*, thereby contributing to the QS feedback loop and virulence of strain OE1‐1.

## Graphical abstract

CbhA is involved in the full expression of *phcA*, encoding a LysR family transcriptional regulator, contributing to the quorum sensing and virulence of *Ralstonia pseudosolanacearum* strain OE1‐1.

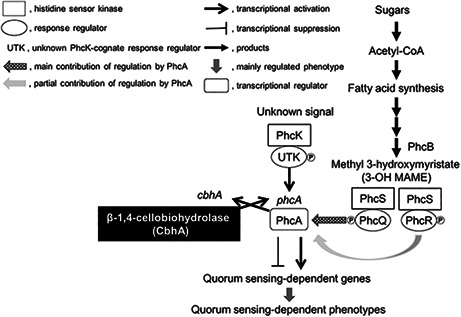

## 1 INTRODUCTION

Bacteria monitor quorum sensing (QS) signals to track changes in abundance and to activate QS for the synchronous control of the expression of genes beneficial for vigorous replication, adaptation to environmental conditions, and virulence (Ham, 2013; Rutherford & Bassler, 2012). The soilborne gram‐negative β‐proteobacterial *Ralstonia solanacearum* species complex (RSSC) is globally distributed under diverse environmental conditions. The RSSC, which infects more than 250 plant species belonging to over 50 families, causes potentially devasting bacterial wilt disease and seriously affects plant production worldwide (Mansfield et al., 2012). The phylotype I *Ralstonia pseudosolanacearum* strain OE1‐1 (Kanda et al., 2003) produces methyl 3‐hydroxymyristate (3‐OH MAME) as a QS signal (Kai et al., 2015; Ujita et al., 2019). This QS signal, which is synthesized by the methyltransferase PhcB, is sensed through the sensor histidine kinase PhcS (Hikichi et al., 2017), leading to the phosphorylation of two regulators, PhcQ and PhcR, that strongly and partially contribute to the regulation of QS‐dependent genes, respectively, via the LysR family transcriptional regulator PhcA (Takemura et al., 2021). The sensor histidine kinase PhcK is required for full expression of *phcA* independently of 3‐OH MAME sensing (Senuma et al., 2020).

RSSC strains in soil invade plant roots through wounds or natural openings from which secondary roots emerge. After colonizing the intercellular spaces of the root cortex and vascular parenchyma, the bacteria eventually enter xylem vessels and spread into the stem and leaves through the xylem (Araud‐Razou et al., 1998; Vasse et al., 1995). Liu et al. (2005) have demonstrated that the virulence of the phylotype I *R*. *pseudosolanacearum* strain GMI1000 involves six plant cell wall‐degrading enzymes (PCWDEs), namely endo‐polygalacturonase, exo‐poly‐α‐galacturonosidase, galacturan 1,4‐α‐galacturonidase, pectin methyl esterase, β‐1,4‐cellobiohydrolase (CbhA), and β‐1,4‐endoglucanase (Egl), all secreted through the type II secretion system (T2SS; Liu et al., 2005). Tsujimoto et al. (2008) have shown that the cell wall degradation activity of PCWDEs secreted through the T2SS is required for the virulence of strain OE1‐1 and its infection of xylem vessels. Furthermore, PhcA positively regulates the expression of *egl* and *cbhA* in strain OE1‐1 in the active QS state (Mori et al., 2018).

Strain OE1‐1 preferentially attaches to the surface of tomato root elongation zones and then colonizes the intercellular spaces between the epidermis and cortex (Inoue et al., 2023). This colonization leads to the degradation of cell walls of cortical cells adjacent to the epidermis. To infect xylem vessels and achieve virulence, strain OE1‐1 then forms mushroom‐shaped biofilms in the degraded cortical cells. The *phcA*‐deletion mutant (Δ*phcA*) and a T2SS‐deficient mutant have no cellulose degradation ability and thus cannot infect cortical cells of tomato roots and exhibit virulence in tomato plants. Compared with strain OE1‐1, the *egl*‐deletion mutant (Δ*egl*) exhibits lower cellulose degradation activity and reduced infectivity in cortical cells, in turn leading to lowered infectivity in xylem vessels and significantly reduced virulence. Cellulose degradation activity is thus believed to be positively corelated with the virulence of *R. pseudosolanacearum* strains. Nevertheless, the function of PCWDEs other than Egl in the infection of tomato roots by strain OE1‐1 remains elusive.

In this study, we aimed to elucidate the function of one PCWDE, CbhA, in the infection of tomato roots by strain OE1‐1. To achieve this goal, we first analysed the cellulose degradation activity and virulence of the *cbhA*‐deletion mutant (Δ*cbhA*). In addition, we examined the alternative function of CbhA in the virulence of strain OE1‐1.

## 2 RESULTS

### 2.1 Cellulose degradation activity of Δ*cbhA*

**FIGURE 1:**
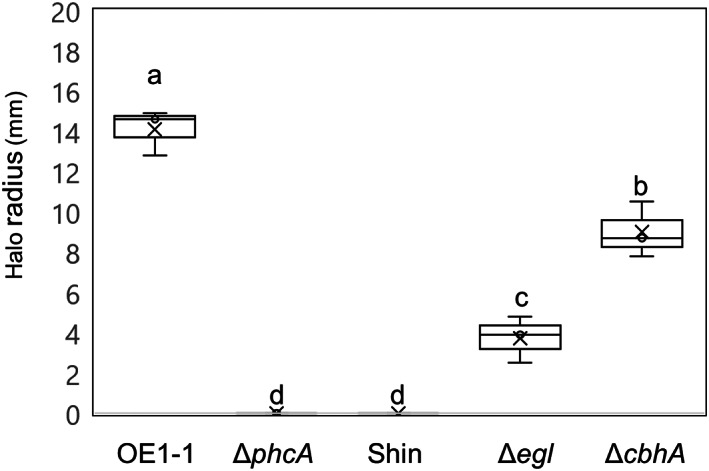
Cellulose degradation activity of *Ralstonia pseudosolanacearum* OE1‐1 and *phcA*‐deletion (Δ*phcA*), type II secretion system‐deficient (Shin), *egl*‐deletion (Δ*egl*), and *cbhA*‐deletion (Δ*cbhA*) mutants. The protein fraction from the supernatant of each *R. pseudosolanacearum* culture strain was placed on carboxy methyl cellulose, and the halos were observed by staining with Congo red. Three biological replicates were tested, with seven technical replicates per biological replicate. Bars represent maximum and minimum values of three biological replicates. Crosses represent average values of three biological replicates. Means were analysed for significant differences between *R. pseudosolanacearum* strains by analysis of variance followed by Tukey–Kramer's HSD test. Statistically significant differences are indicated by different lowercase letters (*p* < 0.05).

To elucidate the cellulose degradation activity of CbhA, we first generated the deletion mutant Δ*cbhA* (Table 1) and compared this mutant with other strains. Two deletion strains, the T2SS‐deficient mutant Shin (Table 1; Tsujimito et al., 2008) and Δ*phcA* (Table 1; Mori et al., 2016), displayed loss of cellulose degradation activity (Figure 1), whereas Δ*egl* (Table 1; Inoue et al., 2023) exhibited significantly reduced cellulose degradation activity compared with strain OE1‐1 (Figure 1; *p* < 0.05). Although Δ*cbhA* had significantly lower cellulose degradation activity compared with strain OE1‐1 (Figure 1; *p* < 0.05), *egl* deletion caused a more significant reduction than did *chbA* deletion (Figure 1; *p* < 0.05). These results suggest that the contribution of CbhA to the cellulose degradation activity of strain OE1‐1 is smaller than that of Egl.

**TABLE 1:** Strains and plasmids used in this study.

	Relevant characteristics	Source
Plasmids		
pK18mobsacB	Km^r^, *oriT* (RP4), *sacB*, *lacZα*	Kvitko and Collmer (2011)
pdelta‐cbhA	pK18mobsacB derivative carrying a 1.1‐kb DNA fragment for *cbhA* deletion, Km^r^	This study
pUC18‐mini‐*Tn*7T‐Gm	Gm^r^	Choi et al. (2005)
pTNS2	Helper plasmid carrying T7 transposase gene	Choi et al. (2005)
pCcbhA	pUC18‐mini‐Tn*7*T‐Gm derivative carrying a 2.8‐kb fragment for *cbhA* complementation, Gm^r^	This study
p0480prophcA	pUC18‐mini‐Tn*7*T‐Gm derivative carrying a 1.6‐kb fragment for constitutive expression of *phcA*, Gm^r^	Senuma et al. (2020)
*Escherichia coli*		
DH5α	*recA1 endA1 gyrA96 thi‐1 hsdR17supE44* Δ(*lac*)*U169*(ϕ*80lac*ΔM15)	Takara Bio
*R. solanacearum*		
OE1‐1	Wild‐type strain, phylotype I, race 1, biovar 4,	Kanda et al. (2003)
Δ*phcA*	*phcA*‐deletion mutant of OE1‐1	Mori et al. (2016)
Shin	Type II secretion system‐deficient mutant of OE1‐1	Tsujimoto et al. (2008)
Δ*cbhA*	*cbhA*‐deletion mutant of OE1‐1	This study
*cbhA*‐comp	A transformant of Δ*cbhA* with pCcbhA containing native *cbhA*, Gm^r^	This study
phcA‐compΔ*cbhA*	A transformant of Δ*cbhA* with p0480prophcA containing the promoter of *RSc0480* gene‐fused native *phcA*, Gm^r^	This study

### 2.2 Virulence of Δ*cbhA* in tomato plants

**FIGURE 2:**
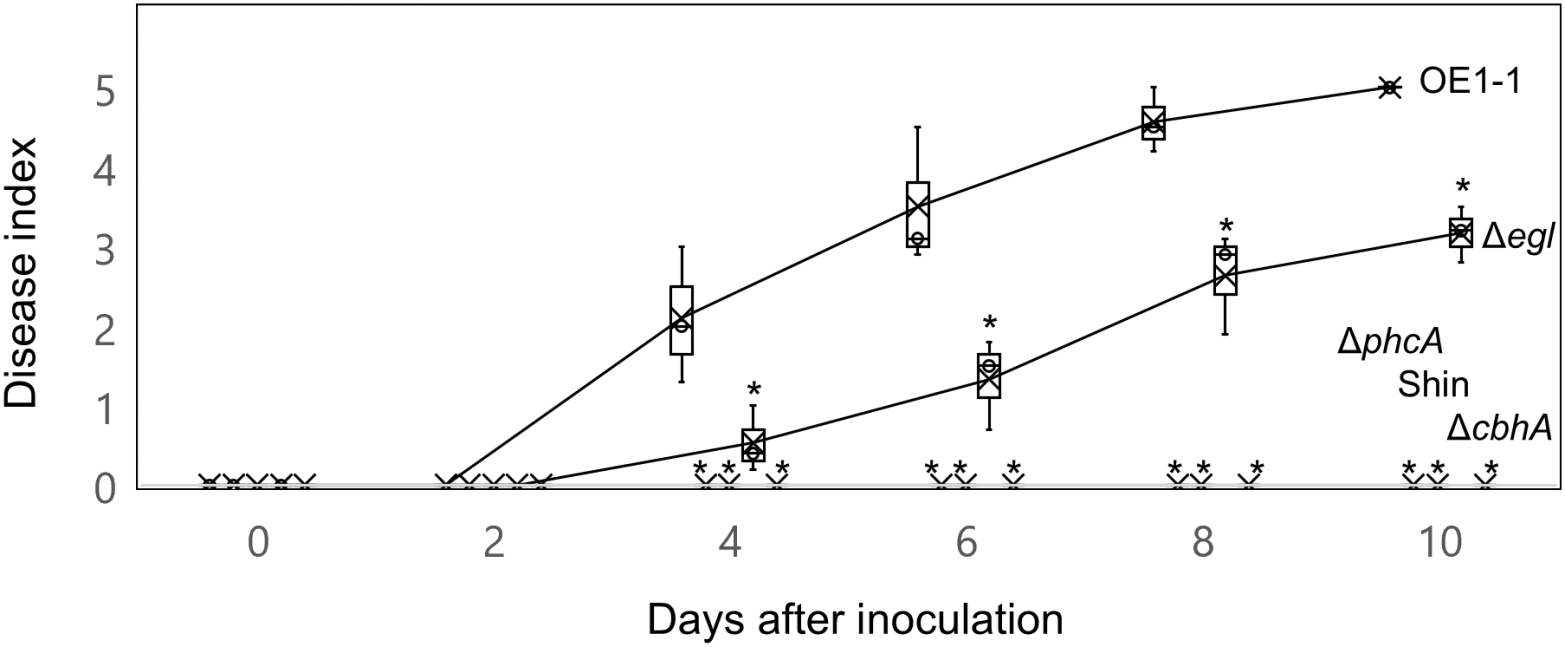
Virulence of *Ralstonia pseudosolanacearum* OE1‐1 and *phcA*‐deletion (Δ*phcA*), type II secretion system‐deficient (Shin), *egl*‐deletion (Δ*egl*), and *cbhA*‐deletion (Δ*cbhA*) mutants. Plants were rated according to the following disease index scale: 0, no wilting; 1, 1%–25% wilting; 2, 26%–50% wilting; 3, 51%–75% wilting; 4, 76%–99% wilting; 5, dead. For each bacterial strain, three independent groups were tested, with 12 technical replicates per group. Bars represent maximum and minimum values of three independent groups. Crosses represent average values of three independent groups. Asterisks indicate values significantly different from those obtained following inoculation with strain OE1‐1 (*p* < 0.05, *t* test).

We next analysed the effects of *cbhA* deletion on *R. pseudosolanacearum* virulence. All tomato plants inoculated with strain OE1‐1 exhibited wilt symptoms and were dead by 10 days after inoculation. Δ*egl* exhibited significantly reduced virulence compared with strain OE1‐1 (*p* < 0.05, *t* test; Figure 2). On the contrary, Δ*cbhA* had lost its virulence, similar to Δ*phcA* and Shin. These results suggest that CbhA, similar to PhcA, is a major contributor to the virulence of strain OE1‐1 in tomato plants.

### 2.3 The behaviour of Δ*cbhA* in tomato roots

**FIGURE 3:**
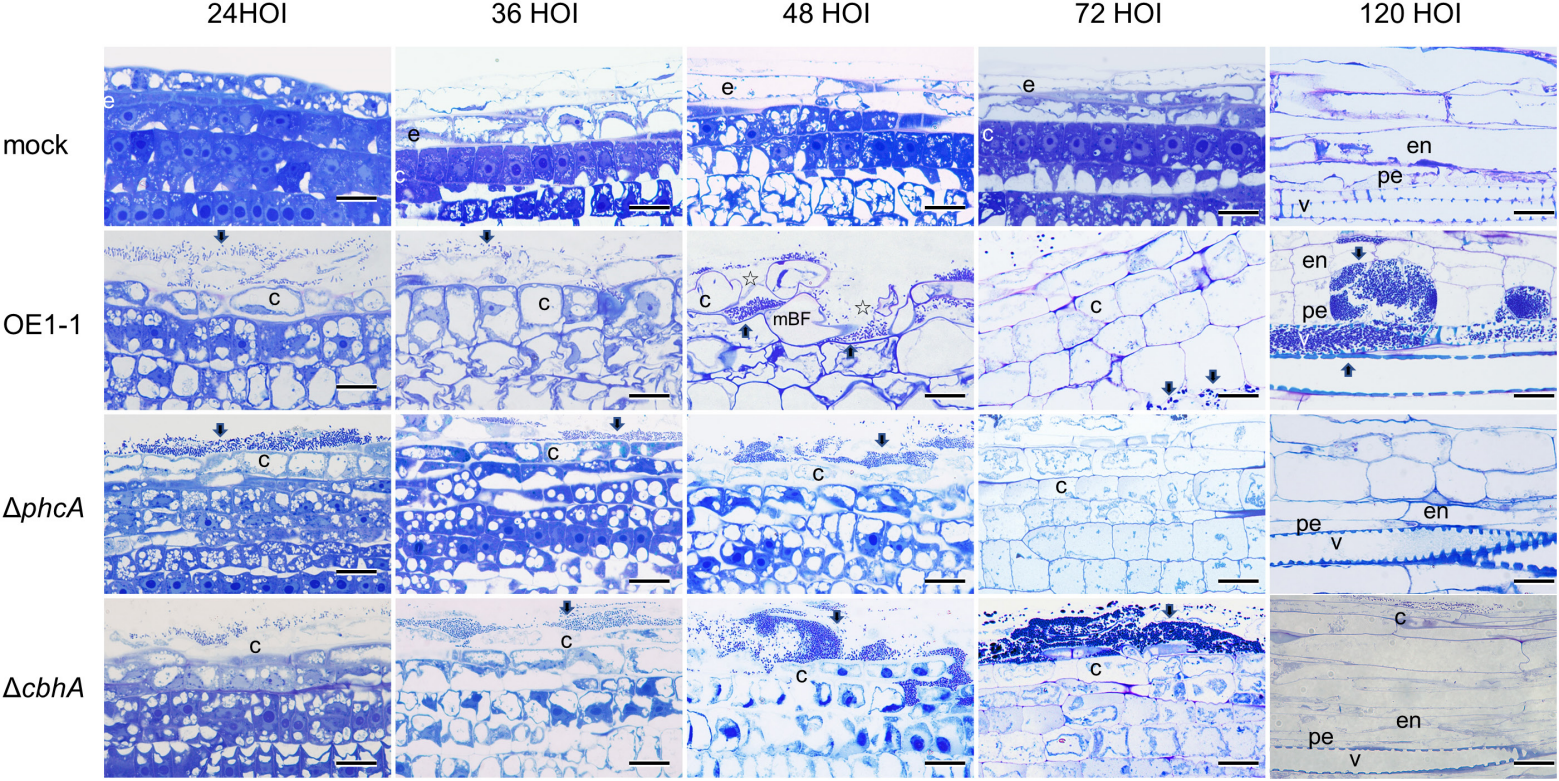
Behaviour of *Ralstonia pseudosolanacearum* OE1‐1 and *phcA*‐deletion (Δ*phcA*) and *cbhA*‐deletion (Δ*cbhA*) mutants in roots of tomato plants. Microscopic observation was carried out on roots of 4‐day‐old tomato seedlings (mock [control] and after 24, 36, 48, 72, and 120 h of incubation [HOI] with *R. pseudosolanacearum* strains). Longitudinal semi‐thin resin sections were stained with toluidine blue. Arrows indicate OE1‐1 cells. Bars indicate 50 μm. e, epidermal cell; c, cortical cell; en, endodermis cell; pe, pericycle cell; v, xylem vessel; mBF, mushroom‐shaped biofilm; ☆, degenerated cortical cell wall.

To observe the behaviour of Δ*cbhA* in tomato roots, we placed 4‐day‐old tomato seedlings with approximately 20‐mm‐long roots on agar plates and added a suspension of *R. pseudosolanacearum* strain OE1‐1, Δ*phcA*, or Δ*cbhA* 10 mm away from the roots (Inoue et al., 2023). Longitudinal semi‐thin (800‐nm) resin sections of the tomato roots were stained with toluidine blue and observed under an optical microscope. After 36 h of co‐incubation (HOI), strain OE1‐1 had infected cell wall‐denatured cortical cells adjacent to the epidermis in the root elongation zone (Figure 3). At the same time point, Δ*phcA* and Δ*cbhA* cells were observed in intercellular spaces between the epidermis and cortex. At 48 HOI, mushroom‐shaped biofilms of strain OE1‐1 were observed in the cell wall‐denatured cortical cells. By contrast, we observed only a few Δ*cbhA* cells but no Δ*phcA* cells in intercellular spaces of the cortex at 48 HOI. Furthermore, neither Δ*phcA* nor Δ*cbhA* cells were observed in cortical cells. At 72 HOI, OE1‐1 cells were found in intercellular spaces between the cortex and endodermis. At 120 HOI, OE1‐1 cells were observed in intercellular spaces of the cortex and endodermis and also in cell wall‐denatured pericycle cells without nuclei and xylem vessels, but no Δ*phcA* or Δ*cbhA* cells were detected in either location. These observations suggest that *cbhA* deletion leads to a loss in infectivity in cortical cells and subsequent loss of infection in xylem vessels and virulence, similar to *phcA* deletion. According to these results, CbhA may have a wider range of roles than just cellulose degradation in OE1‐1 virulence.

### 2.4 RNA‐seq transcriptome analysis of Δ*cbhA*

To identify QS signalling pathways and QS‐dependent genes of strain OE1‐1, we previously performed a transcriptome analysis based on RNA sequencing (RNA‐seq) of *R. pseudosolanacearum* strains grown in quarter‐strength M63 until OD_600_ = 0.3 and then mapped the RNA‐seq reads on the GMI1000 genome. Mapping of RNA‐seq reads of the OE1‐1 strain to the genome of strain GMI1000 (Salanoubat et al., 2002) resulted in the identification of 4370 protein‐coding transcripts (Table S1). To extract genes with significant expression changes, the following thresholds were applied: *q* value < 0.05 and log_2_(fold change) ≥ |2|.

**FIGURE 4:**
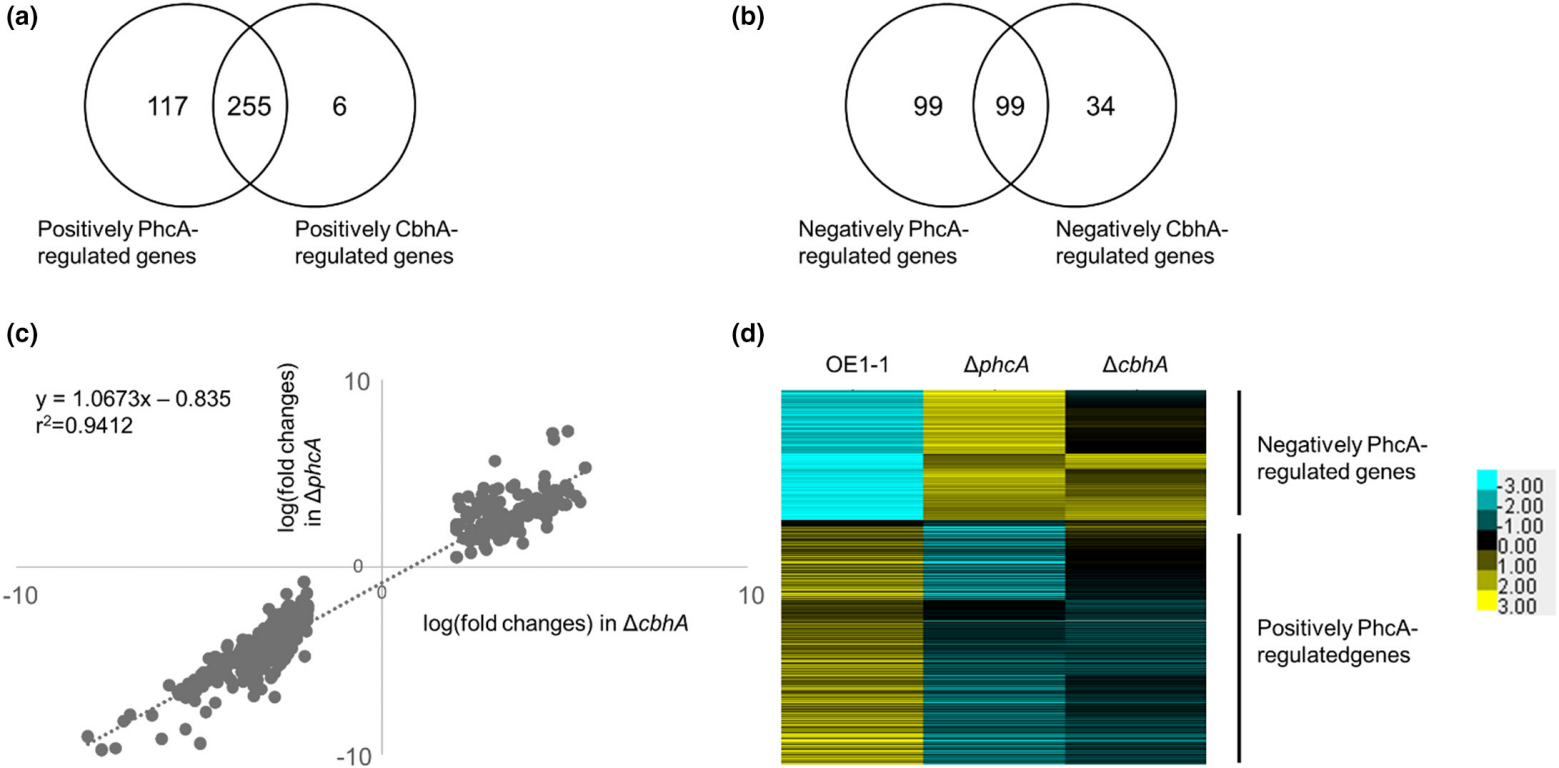
RNA‐seq transcriptome analysis of CbhA‐regulated genes of *Ralstonia pseudosolanacearum* strains grown in quarter‐strength M63 medium until OD_600_ = 0.3. (a) The number of genes exhibiting significantly decreased expression (log_2_(fold change) < −2) in *phcA*‐deletion (Δ*phcA*) and *cbhA*‐deletion (Δ*cbhA*) mutants relative to their expression in strain OE1‐1 (*q* < 0.05). (b) The number of genes exhibiting significantly increased expression (log_2_(fold change) > 2) in Δ*phcA* and Δ*cbhA* relative to their expression in strain OE1‐1 (*q* < 0.05). (c) Correlation of expression levels of CbhA‐regulated genes between *R. pseudosolanacearum* mutants: Δ*phcA* versus mutants. (d) Hierarchical clustering of relative expression levels of PhcA‐regulated genes in *R. pseudosolanacearum* OE1‐1, Δ*phcA*, and Δ*cbhA*. Fragments per kilobase of open reading frame per million fragments mapped values from *R. pseudosolanacearum* OE1‐1, Δ*phcA*, and Δ*cbhA* were normalized prior to analysis of differentially expressed genes.

Compared with their expression levels in OE1‐1, 261 genes in Δ*cbhA* exhibited significantly downregulated expression and were thus inferred to be positively CbhA‐regulated genes, whereas 133 genes in Δ*cbhA* had significantly upregulated expression (i.e., negatively CbhA‐regulated genes) (Figure 4a,b, Table S2). Interestingly, *phcA* was included among the positively CbhA‐regulated genes (Table S3). No significant differences in expression levels of the QS‐related genes *phcB*, *phcK*, and *phcQ* were detected between Δ*cbhA* and strain OE1‐1 (Table S3). We also conducted an RNA‐seq transcriptome analysis of Δ*phcA* and identified 372 positively PhcA‐regulated genes, including *cbhA*, and 198 negatively PhcA‐regulated genes (Figure 4a,b). Among the positively and negatively CbhA‐regulated genes, 255 genes, including the EPS I production‐related gene *epsB*, and 99 genes, including the flagellar biogenesis‐related gene *fliC*, were found to be positively and negatively PhcA‐regulated genes, respectively (Figure 4a,b, Table S2).

A strong positive correlation of the expression of these CbhA‐regulated genes was observed between Δ*cbhA* and Δ*phcA* (*y*, log_2_(fold change) of Δ*phcA*; *x*, log_2_(fold change) of Δ*cbhA*; *y* = 1.0673*x* − 0.8535, *r* ^2^ = 0.9412; Figure 4c).

We next conducted hierarchical clustering of Δ*phcA*, Δc*bhA*, and strain OE1‐1 based on their relative expression levels normalized against those of PhcA‐regulated genes. The expression patterns of approximately 50% of PhcA‐regulated genes in Δ*chbA* were similar to those in Δ*phcA* (Figure 4d).

The transcriptome analysis thus showed that *cbhA* deletion leads to significantly reduced expression of *phcA*, thereby altering the expression of 255 (68.5%) and 99 (50.0%) positively and negatively PhcA‐regulated genes, respectively.

### 2.5 Gene Ontology enrichment analysis in Δ*cbhA*

To characterize the functions of genes affected by *cbhA* deletion, we performed a Gene Ontology (GO) enrichment analysis. The analysis was applied to three gene categories, namely, those whose expression was affected by *phcA* deletion, *cbhA* deletion, or both. Among positively regulated genes, typical GO terms, including “lipopolysaccharide biosynthetic process,” were enriched in 255 genes affected by both genotypes (Table 2). Among negatively regulated genes, GO terms related to bacterial motility, such as “chemotaxis” and “bacterial‐type flagellum‐dependent cell motility,” were enriched in 99 genes with significantly reduced expression in both ∆*cbhA* and ∆*phcA*. Some terms related to bacterial‐type flagella were only enriched in genes with significantly reduced expression in ∆*cbhA*, whereas terms related to “translation” were enriched in ∆*phcA‐*affected genes but not in ∆*cbhA‐*affected genes.

**TABLE 2:** Gene Ontology enrichment analysis of genes significantly affected by *cbhA* deletion.

Category^a^	GO term	*p* value	Fold enrichment
I	Chemotaxis	<10^−41^	28.2
	Signal transduction	<10^−41^	22.6
	Bacterial‐type flagellum‐dependent cell motility	<10^−41^	39.8
II	Structural constituent of ribosome	1.0 × 10^−41^	27.6
	Translation	3.6 × 10^−39^	24.1
	Ribosome	1.8 × 10^−26^	24.5
III	Bacterial‐type flagellum assembly	2.1 × 10^−10^	71.3
	Bacterial‐type flagellum basal body	8.6 × 10^−10^	71.3
	Chemotaxis	1.1 × 10^−9^	34.6
IV	Lipopolysaccharide biosynthetic process	5.8 × 10^−5^	12.7
	Acylphosphatase activity	9.2 × 10^−4^	19.0
	Cellular biosynthetic process	1.6 × 10^−3^	11.4
V	Oxidoreductase activity	2.9 × 10^−3^	3.0
	Integral component of membrane	3.6 × 10^−3^	1.4
	Ion transport	8.0 × 10^−3^	6.5
VI	Transcription *cis*‐regulatory region binding	1.2 × 10^−3^	807.8
	Protein–DNA complex	1.2 × 10^−3^	807.8
	DNA‐binding transcription activator activity	3.1 × 10^−3^	269.3

^a^ I, log_2_(fold change) > 2, *q* < 0.05 in both ∆*phcA* and ∆*cbhA*; II, log_2_(fold change) > 2, *q* < 0.05 in ∆*phcA*; III, log_2_(fold change) > 2, *q* < 0.05 in ∆*cbhA*; IV, log_2_(fold change) < −2, *q* < 0.05 in both ∆*phcA* and ∆*cbhA*; V, log_2_(fold change) < −2, *q* < 0.05 in ∆*phcA*; VI, log_2_(fold change) < −2, *q* < 0.05 in ∆*cbhA*.

### 2.6 QS‐dependent phenotypes of Δ*cbhA*

**FIGURE 5:**
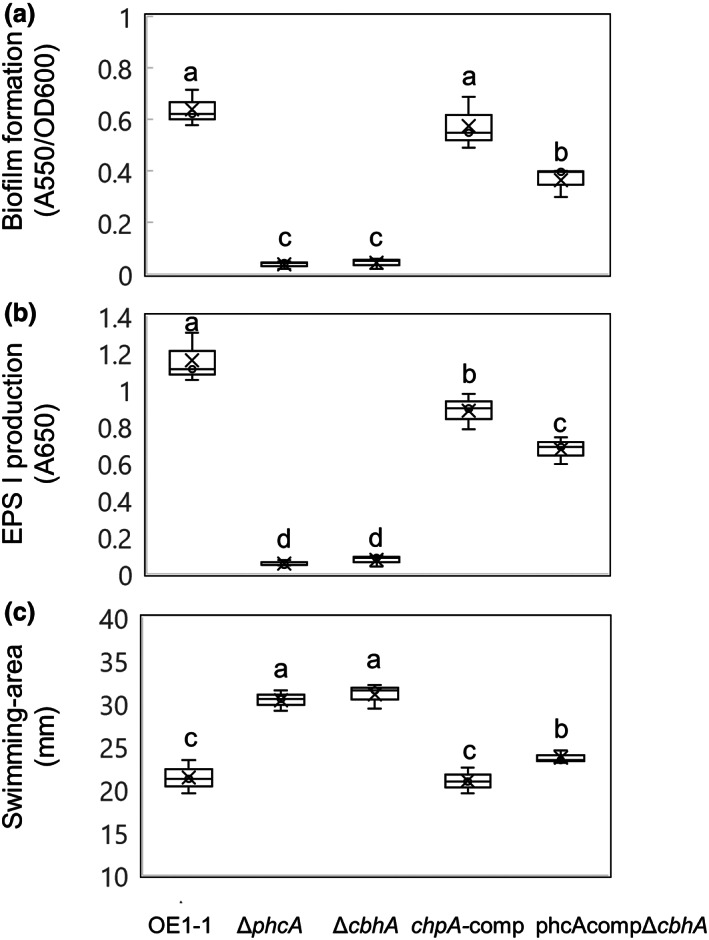
Biofilm formation (a), production of the major exopolysaccharide EPS I (b), and swimming motility (c) of *Ralstonia pseudosolanacearum* OE1‐1 and *phcA*‐deletion (Δ*phcA*) and *cbhA*‐deletion (Δ*cbhA*) mutants, complemented Δ*cbhA* (*cbhA*‐comp), and Δ*cbhA* transformed with *phcA* under the control of a constitutive promoter (phcA‐compΔ*cbhA*). (a) Cells of *R. pseudosolanacearum* were incubated in quarter‐strength M63 medium in wells of polyvinylchloride microtitre plates and stained with crystal violet. Three replicate experiments were conducted using independent samples, with seven technical replicates per experiment. (b) *R. pseudosolanacearum* strains were incubated on quarter‐strength M63 medium solidified with 0.25% agar. Three replicate experiments were conducted using independent samples, with five technical replicates per experiment. (c) *R. pseudosolanacearum* strains were grown on quarter‐strength M63 medium solidified with 0.25% agar. Three biological replicates were conducted, with five technical replicates per biological replicate. Bars represent maximum and minimum values of three biological replicates. Crosses represent average values of three biological replicates. Means were analysed for significant differences between *R. pseudosolanacearum* strains by analysis of variance followed by Tukey–Kramer's HSD test. Statistically significant differences are indicated by different lowercase letters (*p* < 0.05).

To assay the influence of CbhA on QS activity, we first examined the in vitro biofilm formation of Δ*cbhA* and the complemented Δ*cbhA* strain *cbhA*‐comp (Table 1). Similar to Δ*phcA*, Δ*cbhA* exhibited significantly less biofilm formation than did strain OE1‐1 (Figure 5a; *p* < 0.05). In addition, significantly more biofilm formation was observed in strain *cbhA*‐comp than in Δ*cbhA* (*p* < 0.05).

The transcriptome analysis revealed that *cbhA* deletion led to significantly reduced expression levels of *epsB*, a gene related to EPS I production. We next assayed EPS I production by Δ*cbhA* and *cbhA*‐comp. *cbhA* deletion led to significantly reduced production of EPS I (Figure 5b; *p* < 0.05), similar to the effect of *phcA* deletion, whereas strain *cbhA*‐comp produced significantly more EPS I than did Δ*cbhA* (*p* < 0.05).

The transcriptome analysis also indicated that *cbhA* deletion led to significantly enhanced expression levels of the flagellar biogenesis‐related gene *fliC*, which is required for the swimming motility of strain OE1‐1. Compared with strain OE1‐1, Δ*cbhA* exhibited significantly enhanced swimming motility, similar to Δ*phcA* (Figure 5c; *p* < 0.05). Finally, *cbhA*‐comp exhibited significantly reduced swimming motility relative to Δ*cbhA* (*p* < 0.05). Taken together, all of these results suggest that complemented *cbhA* expression leads to recovery of the QS‐dependent phenotypes of Δ*cbhA*.

To analyse whether the QS‐dependent phenotypes of Δ*cbhA* are recovered when the strain is transformed with *phcA* harbouring a QS‐independent, constitutive active promoter of the *RSc0480* gene, we created the transformant phcA‐compΔ*cbhA* (Table 1). The strain phcA‐compΔ*cbhA* exhibited significantly enhanced biofilm formation (Figure 5a; *p* < 0.05) and EPS I production (Figure 5b; *p* < 0.05) compared with Δ*cbhA*. In addition, phcA‐compΔ*cbhA* exhibited significantly reduced swimming motility compared with Δ*cbhA* (Figure 5c; *p* < 0.05). These results suggest that complemented *phcA* expression leads to recovery of the QS‐dependent phenotypes of Δ*cbhA*.

### 2.7 Expression levels of QS‐related genes in Δ*cbhA* in infected tomato roots

**FIGURE 6:**
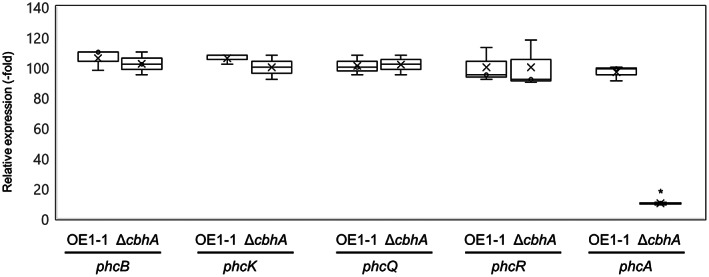
Expression of quorum sensing‐related genes *phcB*, *phcK*, *phcQ*, *phcR*, and *phcA* in *Ralstonia pseudosolanacearum* OE1‐1 and a *cbhA*‐deletion mutant (Δ*cbhA*) after inoculation of 8‐week‐old tomato plants using a root‐dip inoculation procedure. RNA was extracted from the roots of tomato plants 3 days after inoculation as described in the Experimental Procedures section. The *rpoD* gene was used as an internal control for reverse transcription‐quantitative PCR. The RNA levels of the analysed genes are expressed relative to the *rpoD* expression level. The experiments were performed with three biological replicates and two technical replicates. Bars represent maximum and minimum values of three biological replicates. Crosses represent average values of three biological replicates. Asterisks indicate values that are significantly different from those following inoculation with strain OE1‐1 (*p* < 0.05, *t* test).

To analyse the expression level of *phcA* in *R. pseudosolanacearum* strains used to infect tomato roots, 8‐week‐old tomato plants were inoculated with *R. pseudosolanacearum* strains using a root‐dip inoculation procedure. Reverse transcription‐quantitative PCR (RT‐qPCR) was then carried out using total RNA isolated from tomato roots 3 days after inoculation. No significant differences in the expression levels of *phcB*, *phcK*, or *phcQ* were detected between strain OE1‐1 and Δ*cbhA*. By contrast, the expression level of *phcA* was significantly lower in Δ*cbhA* than in strain OE1‐1 (*p* < 0.05, *t* test; Figure 6).

## 3 DISCUSSION

The behaviour of RSSC cells and accompanying morphological changes in tomato cells can be assayed by optical microscopic observation of longitudinal semi‐thin resin sections of tomato roots co‐incubated with an RSSC strain in an in vitro pathosystem (Inoue et al., 2023). Using this system, we previously demonstrated that strain OE1‐1 preferentially attaches to surfaces of tomato root meristematic and elongation zones; it then colonizes intercellular spaces between the epidermis and cortex in the elongation zone of tomato roots to activate QS, thereby infecting the cell wall‐degenerated, nucleus‐containing cortical cell through QS‐induced PCWDEs, such as Egl, secreted through the T2SS (Inoue et al., 2023). This activation leads to subsequent infection of xylem vessels and facilitates the virulence of strain OE1‐1. Liu et al. (2005) have demonstrated that proteins other than PCWDEs that are secreted via the T2SS contribute substantially to the ability of strain GMI1000 to systemically colonize tomato plants. In the present study, Δ*cbhA* exhibited higher cellulose degradation activity compared with Δ*egl* (Figure 1); however, Δ*cbhA* lost its virulence in tomato plants, similar to Δ*phcA*, even though Δ*egl* exhibited weak virulence (Figure 2). Microscopic analysis of the behaviour of Δ*cbhA*, Δ*phcA*, and strain OE1‐1 in roots of tomato seedlings 4 days after sowing indicated that Δ*cbhA* lost its infectivity in cortical cells, thereby preventing its subsequent infection of xylem vessels, similar to Δ*phcA* (Figure 3). These results demonstrate that CbhA is involved in the degradation of cortical cell walls via its cellulose degradation activity as well as through an additional PhcA‐dependent virulence mechanism that influences OE1‐1 infectivity in cortical cells.

The transcriptome analysis indicated that *cbhA* deletion led to significantly reduced expression of *phcA*, which in turn significantly reduced the expression of more than 50% of PhcA‐regulated genes, including not only PCWDE genes, such as *egl*, but also virulence‐related genes, such as *eps* genes, *lecM*, *ralA*, *ralD*, and *xpsR* (Figures 4 and 5; Tables [Link], [Link]). *cbhA* deletion also led to a significant change in QS‐dependent phenotypes (Figure 5). Furthermore, the generation of QS‐dependent phenotypes of Δ*cbhA* transformed with *phcA* under the control of a constitutive promoter demonstrated that reduced expression of *phcA* in Δ*cbhA* is responsible for the change in QS‐dependent phenotypes of Δ*cbhA*. In addition, expression levels of *phcA* in Δ*cbhA* inoculated into tomato plants were significantly lower than those in strain OE1‐1 (Figure 6); furthermore, Δ*cbhA* lost its virulence in tomato plants, similar to Δ*phcA* (Figure 2). We thus infer that *cbhA* deletion leads not only to significantly reduced cellulose activity, but also to significantly reduced expression of *phcA* and subsequent virulence loss.

Transcriptome analysis coupled with RT‐qPCR and RNA‐seq revealed that *cbhA* deletion led to approximately 10% reduced expression of *phcA*. According to the GO enrichment analysis, GO terms “lipopolysaccharide biosynthetic process,” “acylphosphatase activity,” and “cellular biosynthetic process” included in cluster IV were enriched in 255 genes with significantly reduced expression levels in both Δ*phcA* and Δ*cbhA* (Table 2). Furthermore, the GO terms “chemotaxis” and “bacterial‐type flagellum‐dependent cell motility” included in cluster I were enriched in 99 genes with significantly reduced expression levels in both mutants. We thus conclude that the regulation of these genes by PhcA is dependent on the expression level of *phcA*. Although the regulation of genes included in clusters II and V in the GO enrichment analysis was dependent on PhcA, the expression level of *phcA* did not affect their expression levels. The significantly enhanced expression level of some “flagella‐mediated motility‐ and chemotaxis‐related genes” included in cluster III because of the deletion of *cbhA* but not *phcA* suggests that *cbhA* deletion influences other types of transcriptional regulation besides PhcA‐dependent regulation.

Our transcriptome analysis showed that *cbhA* deletion led to significantly reduced expression of *phcA*. One possible idea to explain how *cbhA* contributes to *phcA* regulation is feedback regulation of QS. We previously reported several mechanisms of QS feedback regulation in strain OE1‐1. In the active state of QS, PhcA regulates the expression of QS‐dependent genes responsible for QS‐dependent phenotypes, including virulence, to induce production of virulence‐related aryl‐furanone secondary metabolites, ralfuranones (Kai et al., 2014, 2016), and the major exopolysaccharide EPS I (Genin & Denny, 2012; Schell, 2000). These secondary metabolites are associated with the feedback loop of QS‐dependent gene regulation by PhcA (Hayashi, Senuma, et al., 2019; Mori et al., 2018). In the QS active state, expression of *lecM*, encoding the lectin LecM, is induced, and LecM affects the activation of QS by regulating the stability of extracellularly secreted 3‐OH MAME (Hayashi, Kai, et al., 2019). *cbhA* deletion led to a significant reduction in ralfuranone production‐related *ralA*, EPS I production‐related *epsB* and *lecM*, and *phcA*. This phenomenon may inhibit the feedback loop of QS‐dependent gene regulation by PhcA. In regard to another pathway regulating *phcA*, PhcK is reportedly required for full expression of *phcA* (Senuma et al., 2020). Nevertheless, *cbhA* deletion did not lead to significantly reduced expression levels of *phcK*. Taken together, these results suggest that CbhA contributes to the feedback regulation of QS by positively regulating *phcA* expression independently of PhcK.

In this study, *R. pseudosolanacearum* strains were incubator‐grown in quarter‐strength M63 medium lacking sugars such as cellulose. We thus suspect that carbohydrate metabolites degraded by CbhA are not involved in the regulation of *phcA* expression. However, we have little information on the regulatory mechanisms of *phcA* expression by CbhA. The involvement of PCWDEs, whose production is induced via QS, in virulence has been reported in plant soft‐rotting bacteria as well as the RSSC (Barnard et al., 2007; Reverchon & Nasser, 2013). However, our results are the first observation of the function of PCWDEs involved in the feedback loop in the QS of plant‐pathogenic bacteria. The role of CbhA in the QS‐dependent gene regulation by PhcA elucidated in this study should help provide insight into novel QS feedback mechanisms of CbhA functions and their involvement in the virulence of RSSC members infecting host plants.

## 4 EXPERIMENTAL PROCEDURES

### 4.1 Bacterial strains, plasmids, and growth conditions

*R. pseudosolanacearum* strains (Table 1) were routinely grown in quarter‐strength M63 medium (Cohen & Rickenberg, 1956) at 30°C. *Escherichia coli* strains were grown in lysogeny broth medium (Hanahan, 1983) at 37°C. Selective media contained kanamycin (50 μg/ml) and gentamycin (50 μg/ml).

### 4.2 General DNA manipulations

We used standard techniques (Sambrook et al., 1989) to manipulate DNA and determined DNA sequences using an Automated DNA Sequencer Model 373 (Applied Biosystems). The resulting DNA sequences were analysed using the program DNASYS‐Mac (Hitachi Software Engineering).

### 4.3 Creation of a *cbhA* ‐deletion mutant and complementation constructs

Two fragments were amplified by PCR from the genomic DNA of strain OE1‐1: delta‐cbhA‐1, amplified using primers delta‐cbhA‐1FW (5′‐CGGGATCCCGCGTAGAACATCCGTTCCG‐3′; underlined, *Bam*HI site) and delta‐cbhA‐1RV (5′‐CAGCACCTCACATGCGCGCTGTCAAGGC‐3′), and delta‐cbhA‐2, amplified using primers delta‐cbhA‐2FW (5′‐CGCGCATGTGAGGTGCTGTTTGGGTGTGC‐3′) and delta‐cbhA‐2RV (5′‐CCC*AAGCTT*GCCCGTGAGCGTTATTGGTG‐3′; italics, *Hin*dIII site). The fragment delta‐cbhA amplified using delta‐cbhA‐1FW and delta‐cbhA‐2RV was digested with *Bam*HI and *Hin*dIII and ligated into a *Bam*HI‐ and *Hin*dIII‐digested pK18mobsacB vector (Kvitko & Collmer, 2011) to produce the recombinant plasmid pdelta‐cbhA. The plasmid was electroporated into OE1‐1 competent cells, which were prepared as previously described by Mori et al. (2016). The kanamycin‐sensitive, sucrose‐resistant recombinant Δ*cbhA* (Table 1) was then selected.

The fragment c‐cbhA was amplified by PCR from the genomic DNA of strain OE1‐1 using primers delta‐cbhA‐1FW and delta‐cbhA‐2RV. The generated fragment was then digested with *Bam*HI and *Hin*dIII and ligated into a *Bam*HI‐ and *Hin*dIII‐digested pUC18‐mini‐Tn7‐Gm vector (Choi et al., 2005) to create pCcbhA. pCcbhA was electroporated into Δ*cbhA* with pTNS2 (Choi et al., 2005), and the gentamycin‐resistant strain cbhA‐comp (Table 1) was created.

To analyse the recovered QS‐dependent phenotypes of Δ*cbhA* after transformation with *phcA* under the control of a QS‐independent, constitutive active promoter of the *RSc0480* gene, the recombinant plasmid p0480prophcA (Table 1; Senuma et al., 2020) carrying the promoter of the *RSc0480* gene and *phcA* based on pUC18‐mini‐Tn7T‐Gm was inserted into Δ*cbhA* to create the transformant phcA‐compΔ*cbhA* (Table 1).

### 4.4 Cellulose degradation activity

Plate assays for extracellular cellulase were conducted as described by Tsujimoto et al. (2008). In brief, bacterial strains were grown in PY medium (Kanda et al., 2003) until an OD_600_ of 1.0 was reached, and after lysis, 1 mL of lyzed cell solution was centrifuged at 15,000 × *g* for 5 min to remove cell debris. The protein solution was fractionated from the supernatant using Amicon Ultra‐15 centrifugal filter devices (Merck Millipore). Next, 50‐μL aliquots of the protein fraction were placed in wells created with a cork borer in plates containing 0.1% carboxy methyl cellulose. The wells were incubated at 30°C for 20 h, flooded with 1 mg/mL Congo red, and then examined for halos. Three biological replicates were tested, with seven technical replicates per biological replicate. Means were analysed for significant differences between *R. pseudosolanacearum* strains by analysis of variance (ANOVA) followed by Tukey–Kramer's honestly significant difference (HSD) test.

### 4.5 Virulence assays

First, 8‐week‐old tomato plants (*Solanum lycopersicum* ‘Ohgata‐Fukuju’) were inoculated with *R. pseudosolanacearum* strains (10^8^ cfu/mL) using a root‐dip inoculation procedure as previously described (Hayashi, Kai, et al., 2019). Plants were monitored daily for wilting symptoms, which were rated according to the following disease index scale: 0, no wilting; 1, 1%–25% wilting; 2, 26%–50% wilting; 3, 51%–75% wilting; 4, 76%–99% wilting; 5, dead. For each bacterial strain, three independent groups were tested, with 12 technical replicates per group. The assay means were analysed for significant differences between *R. pseudosolanacearum* strains using Student's *t* test in Microsoft Excel.

### 4.6 Microscopic observation of tomato root sections

Seeds of tomato (*S. lycopersicum* ‘Ohgata‐Fukuju’) plants were sterilized with 70% ethanol solution for 20 s and 1% sodium hypochlorite solution for 20 min (Inoue et al., 2023). The seeds were sown on 1% agar in Petri dishes at 30°C under dark and axenic conditions. Four days after sowing, seedlings with approximately 20‐mm‐long roots were placed on agar plates, and 200 μL of OE1‐1 suspension at 10^8^ cfu/mL was added to the centre of the plate, 10 mm away from the tomato roots. The tomato seedlings were then incubated at 30°C under dark and axenic conditions. Root pieces were excised 4 mm from the tip, fixed in half‐strength Karnovsky's fixative overnight at 4°C, and then post‐fixed in 1% osmium tetroxide for 1 h at room temperature (Karnovsky, 1965). The specimens were dehydrated in an ethanol series (three times at 30%, 50%, 70%, 80%, 90%, and 100%) for 10 min each at 25°C and then embedded in Quetol‐812 resin (Nisshin EM). Serial semi‐thin sections (800 nm thick) were cut with a diamond knife, mounted on glass slides, stained with toluidine blue, and observed under a BX53 optical microscope equipped with a DP74 camera (Olympus).

### 4.7 Transcriptome analysis based on RNA‐seq

Total RNA was extracted from *R. pseudosolanacearum* strains grown in quarter‐strength M63 until OD_600_ = 0.3 with a High Pure RNA Isolation kit (Roche Diagnostics). Ribosomal RNA was eliminated from the extracted total RNA using a Ribo‐Zero rRNA Removal kit (gram‐negative bacteria; Illumina) as previously described (Hayashi et al., 2019). Oriented, paired‐end RNA‐seq (2 × 100 bp) was performed on HiSeq 2000 (Illumina) and DNBSEQ‐G400 (MGI Tech) systems by Bioengineering Lab Co. (Sagamihara, Japan). The generated reads were trimmed with Cutadapt (v. 1.1; http://code.google.com/p/cutadapt/) and Trimmomatic (v. 0.32; http://www.usadellab.org/cms/?page=trimmomatic) and then mapped with the TopHat program (v. 2.0.10; http://tophat.cbcb.umd.edu/) on the GMI1000 strain genome. Three independent biological replicates were sequenced per strain.

### 4.8 Differential gene expression analysis

Statistical analysis of the RNA‐seq data was performed in the R environment. Genes with zero counts in at least one OE1‐1 sample were excluded. The RNA‐seq read counts of the remaining genes were normalized using the function calcNormFactors (trimmed mean of M value normalization) in the package edgeR (Robinson et al., 2010). To extract genes with significant expression changes, the following thresholds were applied: *q* < 0.05 and log_2_(fold change) ≥ |2|. The false discovery rate (Storey's *q* value) was calculated in edgeR from Benjamini–Hochberg‐corrected *p* values. Hierarchical clustering of all normalized mean expression values based on their relative expression (counts per million) was performed using Cluster 3.0 software (de Hoon et al., 2004). The average value of three replicates per strain was used. Heatmaps were created in TreeView (Eisen et al., 1998). For GO enrichment analysis, GO terms were obtained from QuickGO (https://www.ebi.ac.uk/QuickGO/), and the analysis was performed with the R package GoSeq (Young et al., 2010).

### 4.9 QS‐dependent phenotypes

We examined the in vitro biofilm formation of *R. pseudosolanacearum* strains grown without shaking in quarter‐strength M63 as previously described (Mori et al., 2016). Biofilm formation was quantified based on the absorbance at 550 nm. The resulting value was normalized according to the number of cells (OD_600_). Three biological replicates were included, with five technical replicates per biological replicate. Means were analysed for significant differences between *R. pseudosolanacearum* strains by ANOVA followed by Tukey–Kramer's HSD test.

EPS I production by *R. pseudosolanacearum* cells grown on quarter‐strength M63 solidified with 1.5% agar was quantitatively analysed by ELISA (Agdia) as previously described (Mori et al., 2016). EPS I productivity was quantified according to A_650_ values. Three biological replicates were included, with five technical replicates per biological replicate. Means were analysed for significant differences between *R. pseudosolanacearum* strains by ANOVA followed by Tukey–Kramer's HSD test.

For the swimming motility assay, overnight cultures of *R*. *pseudosolanacearum* strains were washed with distilled water and then diluted to a cell density of 5 × 10^5^ cfu/mL, and 5‐μL aliquots of the cell suspensions were added to the centre of plates containing quarter‐strength M63 medium solidified with 0.25% agar. The swimming‐area diameters of *R. pseudosolanacearum* strains were measured at 48 h postinoculation (Mori et al., 2018). Three biological replicates were conducted, with five technical replicates per biological replicate. Means were analysed for significant differences between *R. pseudosolanacearum* strains by ANOVA followed by Tukey–Kramer's HSD test.

### 4.10 RT‐qPCR‐based quantification of expression levels of QS‐related genes in *R. pseudosolanacearum* strains in infected tomato plants

First, 8‐week‐old tomato (*S. lycopersicum* ‘Ohgata‐Fukuju’) plants were inoculated with *R. pseudosolanacearum* strains (10^8^ cfu/mL) using a root‐dip inoculation procedure as previously described (Hayashi, Kai, et al., 2019). Three days after inoculation, total RNA was isolated from tomato roots with RNAiso (Takara), and RNA samples were treated with DNase I (RNase‐free; Takara). Reverse transcription was carried out with 1 μg total RNA using a PrimeScript RT reagent kit (Takara). qPCR amplifications were carried out in 20‐μL reaction mixtures containing 1 μL of the cDNA stock and 0.4 μL of the appropriate primers (10 pM; Table S4) using the SYBR GreenER qPCR reagent system (Invitrogen) on an Applied Biosystems 7300 real‐time PCR system. All values were normalized against the expression level of *rpoD*, which was used as an internal standard for each cDNA sample. No significant differences in *rpoD* expression levels were observed among *R. pseudosolanacearum* strains. The experiments were performed with three biological replicates and two technical replicates. Means of the assays were analysed for significant differences between *R. pseudosolanacearum* strains using Student's *t* test in Microsoft Excel.

## CONFLICT OF INTEREST STATEMENT

The authors declare that they have no conflicts of interest.

## Supporting information


**Table S1** RNA‐seq data for transcripts of PhcA‐regulated genes from *Ralstonia pseudosolanacearum* OE1‐1 and *phcA*‐deletion (Δ*phcA*) and *cbhA*‐deletion (Δ*cbhA*) mutants grown in quarter‐strength M63 medium as well as predicted functions of proteins encoded by the genes.

**Table S2** RNA‐seq data for transcripts of CbhA‐regulated genes in *Ralstonia pseudosolanacearum* OE1‐1 and *phcA*‐deletion (Δ*phcA*) and *cbhA*‐deletion (Δ*cbhA*) mutants grown in quarter‐strength M63 medium as well as predicted functions of proteins encoded by the genes.

**Table S3** RNA‐seq data for transcripts of quorum sensing‐related genes in *Ralstonia pseudosolanacearum* OE1‐1 and *phcA*‐deletion (Δ*phcA*) and *cbhA*‐deletion (Δ*cbhA*) mutants grown in quarter‐strength M63 medium as well as predicted functions of proteins encoded by the genes.

**Table S4** Primers used in the reverse transcription‐quantitative PCR assays.

## Data Availability

The data that support the findings of this study are available from the corresponding author upon reasonable request. RNA‐seq data are available in the NCBI SRA repository at www.ncbi.nlm.nih.gov/sra under accession codes DRR438005, DRR438006, and DRR438007 (WT: OE1‐1); DRR438008, DRR438009, and DRR438010 (∆*phcA*); and DRR438011, DRR438012, and DRR438013 (∆*cbhA*).
