# Supplementary material for: β‐1,4‐Cellobiohydrolase is involved in full expression of phcA, contributing to the feedback loop in quorum sensing of Ralstonia pseudosolanacearum strain OE1‐1
Source: Mol Plant Pathol. 2023 Mar 13;24(6):549–59. doi: 10.1111/mpp.13322 (PMC10189760; doi:10.1111/mpp.13322)
Supplement: Supplementary file 4 — Table S4 Primers used in the reverse transcription‐quantitative PCR assays. [file MPP-24-549-s002.docx]

**Table S4** Primers used in the quantitative real-time polymerase chain reaction assays

| Genes | primers | Nucleotide sequences |
| --- | --- | --- |
| *rpoD*  *phcB*  *phcA*  *phcK*  *phcR*  *phcQ* | rpoD-FW  rpoD-RV  phcB-FW3-514  phcB-RV3-1011  phcA-FW5  phcA-RV5  phcK-FW  phcK-RV  phcR-FW  phcR-RV  phcQ-FW  phcQ-RV | 5ʹ-ATCGTCGAGCGCAACATCCC-3ʹ  5ʹ-AGATGGGAGTCGTCGTCGTCGTG-3ʹ  5ʹ-TACAAGATCAAGCACTACCTCGACTG-3ʹ  5ʹ-GTGCTGTACGCCATCCATCTC-3ʹ  5ʹ-ATGCGTTCCAATGAGCTGGAC-3ʹ  5ʹ-AGATCCTTCATCAGCGAGTTGAC-3ʹ  5ʹ-TGTCGATGTGGCTGCTGATC-3ʹ  5ʹ-CGTTGAACAGGAAATGCGGTTC-3ʹ  5ʹ-GCTGTCGACCTTCCTGAATTC-3ʹ  5ʹ-AAAACGCGGATCAGGTACGG-3ʹ  5ʹ-ATCCTGACCACCGCCTAC-3ʹ  5ʹ-CGACACCGCTAGGTACAG-3ʹ |
